# A nomogram based on inflammation and nutritional biomarkers for predicting the survival of breast cancer patients

**DOI:** 10.3389/fendo.2024.1388861

**Published:** 2024-08-07

**Authors:** Caibiao Wei, Huaying Ai, Dan Mo, Peidong Wang, Liling Wei, Zhimin Liu, Peizhang Li, Taijun Huang, Miaofeng Liu

**Affiliations:** ^1^ Department of Clinical Laboratory, Guangxi Medical University Cancer Hospital, Nanning, China; ^2^ Department of Injection Room, The People’s Hospital of Yingtan, Yingtan, Jiangxi, China; ^3^ Department of Breast, Guangxi Zhuang Autonomous Region Maternal and Child Health Care Hospital, Nanning, China; ^4^ Department of Anesthesiology, First Affiliated Hospital of Guangxi Medical University, Nanning, China

**Keywords:** breast cancer, inflammation, nutrition, nomogram, prognosis

## Abstract

**Background:**

We aim to develop a new prognostic model that incorporates inflammation, nutritional parameters and clinical-pathological features to predict overall survival (OS) and disease free survival (DFS) of breast cancer (BC) patients.

**Methods:**

The study included clinicopathological and follow-up data from a total of 2857 BC patients between 2013 and 2021. Data were randomly divided into two cohorts: training (n=2001) and validation (n=856) cohorts. A nomogram was established based on the results of a multivariate Cox regression analysis from the training cohorts. The predictive accuracy and discriminative ability of the nomogram were evaluated by the concordance index (C-index) and calibration curve. Furthermore, decision curve analysis (DCA) was performed to assess the clinical value of the nomogram.

**Results:**

A nomogram was developed for BC, incorporating lymphocyte, platelet count, hemoglobin levels, albumin-to-globulin ratio, prealbumin level and other key variables: subtype and TNM staging. In the prediction of OS and DFS, the concordance index (C-index) of the nomogram is statistically greater than the C-index values obtained using TNM staging alone. Moreover, the time-dependent AUC, exceeding the threshold of 0.7, demonstrated the nomogram’s satisfactory discriminative performance over different periods. DCA revealed that the nomogram offered a greater overall net benefit than the TNM staging system.

**Conclusion:**

The nomogram incorporating inflammation, nutritional and clinicopathological variables exhibited excellent discrimination. This nomogram is a promising instrument for predicting outcomes and defining personalized treatment strategies for patients with BC.

## 1 Introduction

Breast cancer (BC) is the most frequently diagnosed cancer and is associated with one of the highest mortality rates among female malignant tumors (1). It is estimated that 287,000 new cases of BC will be diagnosed in 2023 (2). The current treatment modalities for BC encompass surgery, chemotherapy, hormonal therapy, and radiation therapy (3, 4). Despite remarkable advancements in early detection and therapeutic approaches, BC patients have poor prognoses (5, 6).

Although the traditional TNM staging system of the American Joint Committee on Cancer (AJCC) is extensively used for predicting prognosis and guiding clinical management, its ability to accurately identify patients at high risk of cancer-related mortality may be limited (7, 8). This limitation arises from the inherent heterogeneity observed in BC, where patients sharing the same stage can exhibit diverse clinical outcomes (9, 10). Patients with BC at the same stage may have different outcomes due to the heterogeneity of the disease (11). Consequently, there exists a critical need to identify reliable and easily applicable predictive models that can complement the TNM staging system and offer more precise predictions of individual patient outcomes.

Extensive Research consistently demonstrates the link between systemic inflammation and poor prognosis in cancer patients (12, 13). Chronic inflammation drives tumor progression, angiogenesis, and metastasis while suppressing the immune response (14, 15). In parallel, the significance of nutritional status in cancer patients’ survival has been widely recognized (16, 17). Cancer-associated malnutrition weakens immune function and triggers inflammation, worsening treatment outcomes (18). These findings underscore the importance of considering both systemic inflammation and nutritional status in the management and prognosis of cancer patients.

Recent studies have shed light on the prevalence of cancer-associated systemic inflammation and malnutrition in the majority of patients with malignancy, including those with BC (14, 19, 20). These factors have been closely linked to tumor progression and have been shown to have a detrimental impact on patient’s clinical outcomes (12, 21, 22). Various inflammation-based and nutritional markers, such as neutrophil (NEU), lymphocyte (LYM), platelet count (PLT), serum ferritin (SF) and lymphocyte-to-monocyte ratio (LMR), as well as prognostic nutritional index hemoglobin (HGB), albumin (ALB), transferrin (TRF), albumin to globulin (AGR), and prealbumin (PA), have been identified as promising clinical prognostic predictors for various cancers due to their simplicity and cost-effectiveness (23–29). However, it is worth noting that many nomogram studies in the literature often fail to consider the assessment of hematological markers encompassing both inflammation and nutritional biomarkers in conjunction with tumor characteristics (30–32). There are two studies of prognostic scoring systems for patients with BC, published by Hua X, et al. (33) and Jiang C, et al. (34). The scoring system developed from those studies provides useful tools for clinicians and researchers to predict the prognostic value of BC patients. However, two of the studies simply classified patients as early-stage or underwent neoadjuvant chemotherapy BC patients, their clinical application is restricted to a subset of patients, not all females with BC treated at their center. Meanwhile, both were developed using limited sample sizes. Furthermore, these models have not received any independent validation, most likely because of the small sample size. In addition, most current studies have primarily focused on the combination of one or a few inflammatory and nutritional parameters with clinicopathological factors, without incorporating more accurate variables such as TNM staging, subtype, and tumor size (35–37). These factors are of great importance in the treatment of BC. Therefore, the assessment of hematological markers including inflammation and nutritional biomarkers, could be of great importance in revealing the survival of patients with BC, which most nomogram studies did not mention.

In this study, our objective was to develop an inexpensive, trustworthy, and more accurate prognostic model by simultaneously combining inflammation and nutritional biomarkers collected from a substantial cohort of nearly 3000 patients diagnosed with BC. By incorporating these diverse factors, we aimed to enhance the accuracy and reliability of prognosis analysis in BC patients.

## 2 Materials and methods

### 2.1 Study population

**Figure 1:**
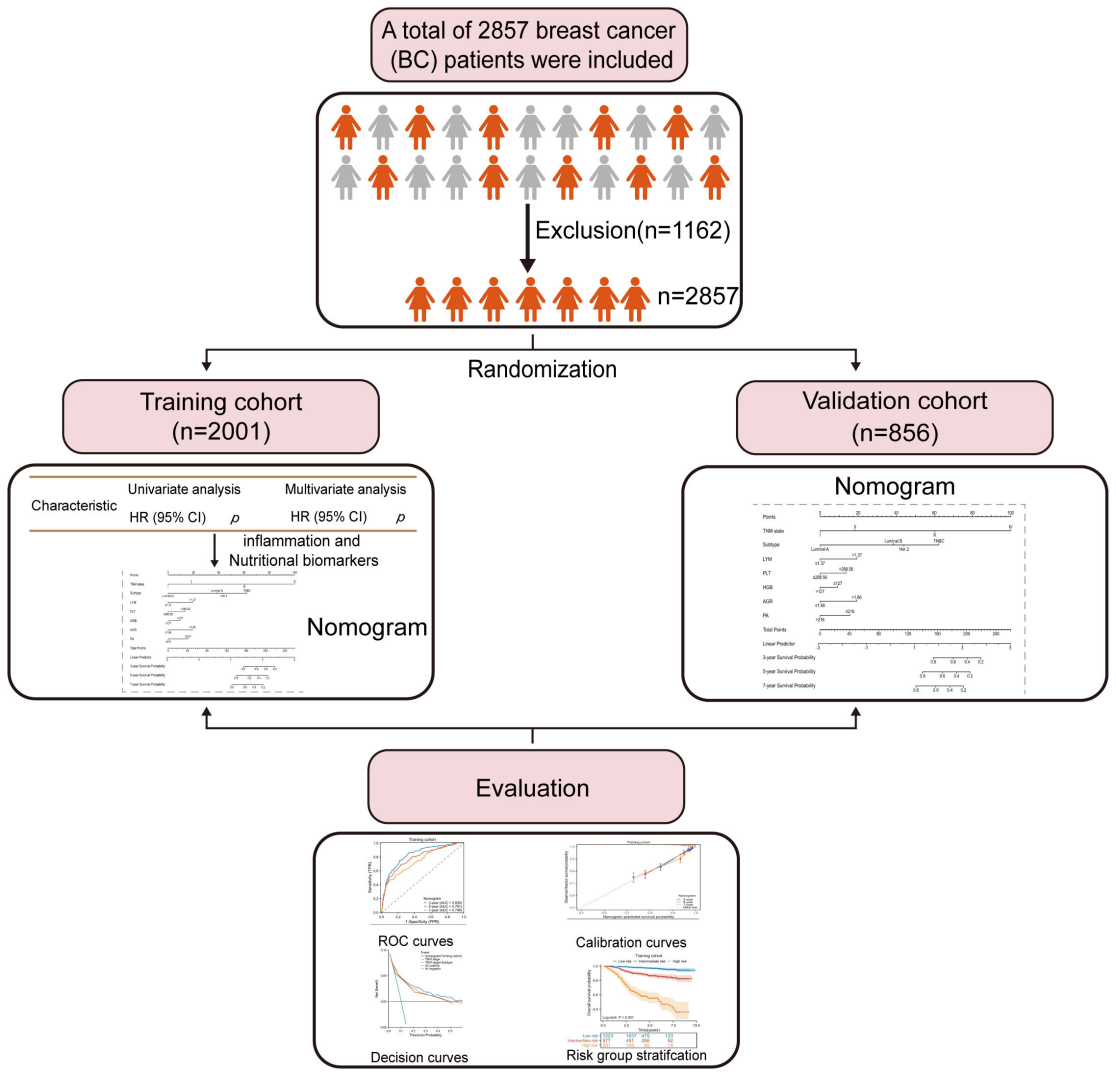
Flow diagram of enrolled participants and evaluation process.

This study included a total of 2857 patients who were diagnosed with BC at Guangxi Medical University Cancer Hospital between 2013 and 2021. Inclusion criteria for the study required: (1) confirmation of the pathological diagnosis of BC; (2) no preoperative chemotherapy or other radiation therapy; (3) absence of acute infections or other inflammatory conditions in the two weeks preceding surgery; (4) availability of complete follow-up information and clinical data; (5) availability of peripheral blood hematological markers before treatment. Patients were removed from the study if any of the following conditions were met: (1) receipt of relevant antitumor therapy (e.g., chemotherapy, radiotherapy) (n=889); (2) lack of clear and definite pathological diagnosis and medical history information (n=96); (3) presence of other malignant tumors except for BC or distant metastasis(n=63); (4) diagnosis of autoimmune diseases or chronic inflammatory conditions (n=79); (5) relapse or *de novo* BC(n=35). All included patients were divided into two groups, with a ratio of 7:3, resulting in a training cohort of 2001 patients and a validation cohort of 856 patients. **Figure 1** depicts the comprehensive workflow for patient selection.

### 2.2 Ethics approval and consent to participate

Our study was approved by the Guangxi Medical University Cancer Hospital Ethical Review Committee (Approve No.LW2023087), Guangxi Zhuang Autonomous Region Maternal and Child Health Care Hospital Ethical Review Committee (Approve No. 6–1, 2024) and conducted following the ethical principles outlined in the Helsinki Declaration of 1964 and its subsequent amendments or other ethical standards with equivalent requirements. To ensure patient confidentiality, the identities of the individuals included in this study were anonymized using computer-generated ID numbers. On admission, all patients provided written consent for their anonymized medical data to be analyzed and published for research purposes.

### 2.3 Data acquisition

In this study, we collected a comprehensive set of clinicopathological, demographic, and laboratory data from 2857 BC patients. Clinicopathological data included the patient’s age, tumor size, histologic type, grade, subtype, and clinical TNM stage based on the most recent AJCC staging system (8th edition) (38), as well as outcomes such as mortality. Pre-treatment inflammation and nutritional biomarkers included the levels of NEU, LYM, MON, PLT, SF, HGB, ALB, TRF, TP, AGR and PA. To facilitate analysis, we also transformed certain clinicopathological characteristics into categorical variables. Furthermore, we calculated several inflammation-related ratios, such as NLR, PLR, and LMR, based on their known associations with the outcomes of interest.

### 2.4 Patient follow-up

We conducted follow-up assessments using a combination of phone interviews and an outpatient surveillance system. The median follow-up time was 54 months (range: 52–55 months). Our primary endpoint of interest was overall survival (OS), which was defined as the duration between the date of surgery and the occurrence of death from any cause or the date of the last follow-up, whichever came first. The period of disease-free survival (DFS) was measured from the date of diagnosis until the occurrence of any recurrence or death. The follow-up period for our study extended until December 2022, or until the date of a patient’s death if it transpired earlier.

### 2.5 Nomogram development and validation

Receiver operating characteristic (ROC) curves were employed to determine the optimal cutoff points for plasma/serum biomarkers using MedCalc software. Statistical analysis was performed using R software version 4.2.1 and SPSS 23.0. The relevance of clinicopathologic characteristics between the training and validation cohorts was analyzed using the chi-square test or Fisher’s exact test. Survival curves were plotted using the Kaplan-Meier method, and differences between groups were assessed using the log-rank test. Univariate and multivariate Cox regression analyses were conducted to identify factors influencing OS. Hazard ratios (HRs) and 95% confidence intervals (CIs) were used to assess the association between patients’ indices and prognosis. In the multivariate Cox regression analysis, only variables with a significance level of *p* < 0.05 in the univariate analysis were included.

A nomogram was constructed using the training cohort of 2001 BC patients based on significant predictors identified through multivariable Cox regression analysis, utilizing R software with the survival and rms packages. The performance of the nomograms was evaluated using the concordance index (C-index), time-dependent receiver operating characteristic (ROC) curve, and the area under the curve (AUC). The accuracy of the model was assessed using calibration plots to compare the predicted and actual OS and DFS. Additionally, decision curve analysis (DCA) was conducted to determine the clinical usefulness of the nomograms by quantifying the net benefits at different threshold probabilities. Results with *p* < 0.05 were considered statistically significant.

### 2.6 External validation of the nomogram

We further validated the feasibility of our model by using BC patients from the Guangxi Zhuang Autonomous Region Maternal and Child Health Care Hospital as an external validation cohort. For external validation of the model, we utilized TNM stage, subtype, LYM, PLT, HGB, AGR, and PA indicators, along with their respective cutoff values, to construct the model in independent cohorts, aiming to assess the robustness and applicability of the model in this study.

## 3 Results

### 3.1 Clinicopathologic characteristics

A total of 2001 patients from the training cohort and 856 patients from the validation cohort were included in our analyses. The demographic and clinical characteristics of the patients are summarized in **Table 1**. No significant differences were observed between the primary and validation cohorts, except for age, LYM, LMR, ALB level, and TRF level. The median follow-up times for the primary and validation cohorts were 55.0 months (range: 53.0 to 57.0 months) and 54.0 months (range:52.0 to 56.0 months), respectively.

**Table 1:** Characteristics of training cohort and validation cohort.

Characteristic	All patients	Training cohort	Validation cohort	*p*
	No. (%)	No. (%)	No. (%)	
**Total**	2857	2001	856	
**Age (years)**				**0.024**
≤52	1910(66.9%)	1364(68.2%)	546(63.8%)	
>52	947(33.1%)	637(31.8%)	310(36.2%)	
**Histologic type**				0.545
IDC	2121(74.2%)	1490(74.5%)	631(73.7%)	
ILC	76(2.7%)	49(2.4%)	27(3.2%)	
Others	660(23.1%)	462(23.1%)	198(23.1%)	
**Grade**				0.906
I	419(14.7%)	290(14.5%)	129(15.1%)	
II	1265(44.3%)	886(44.3%)	379(44.3%)	
III	1173(41.0%)	825(41.2%)	348(40.6%)	
**Subtype**				0.363
Luminal A	402(14.1%)	292(14.6%)	110(12.9%)	
Luminal B	1760(61.6%)	1217(60.8%)	543(63.4%)	
Her 2	391(13.7%)	283(14.1%)	108(12.6%)	
TNBC	304(10.6%)	209(10.5%)	95(11.1%)	
**TNM stage**				0.834
I	644(22.5%)	451(22.5%)	193(22.5%)	
II	1525(53.4%)	1076(53.8%)	449(52.5%)	
III	539(18.9%)	369(18.4%)	170(19.9%)	
IV	149(5.2%)	105(5.3%)	44(5.1%)	
**Tumor size(cm)**				0.255
≤2	912(31.9%)	652(32.6%)	260(30.4%)	
>2	1945(68.1%)	1349(67.4%)	596(69.6%)	
**PLT (10^9^/L)**				0.478
≤288.56	1734(60.7%)	1223(61.2%)	511(59.7%)	
>288.56	1123(39.3%)	778(38.8%)	345(40.3%)	
**MON (10^9^/L)**				0.361
≤0.39	1690(59.2%)	1195(59.7%)	495(57.8%)	
>0.39	1167(40.8%)	806(40.3%)	361(42.2%)	
**NEU (10^9^/L)**				0.484
≤4.84	2254(78.9%)	1586(79.3%)	668(78.0%)	
>4.84	603(21.1%)	415(20.7%)	188(22%)	
**LYM (10^9^/L)**				**<0.001**
≤1.37	471(16.5%)	295(14.7%)	176(20.6%)	
>1.37	2386(83.5%)	1706(85.3%)	680(79.4%)	
**PLR (10^9^/L)**				0.322
≤196.4	2316(81.1%)	1632(81.6%)	684(79.9%)	
>196.4	541(18.9%)	369(18.4%)	172(20.1%)	
**LMR (10^9^/L)**				**<0.001**
≤4.92	1187(41.5%)	907(45.3%)	280(32.7%)	
>4.92	1670(58.8%)	1094(54.7%)	576(67.3%)	
**NLR (10^9^/L)**				0.932
≤1.69	988(34.6%)	691(34.5%)	297(34.7%)	
>1.69	1869(65.4%)	1310(65.5%)	559(65.3%)	
**ALB (g/L)**				**0.005**
≤44.6	2382(83.4%)	1643(82.1%)	739(86.3%)	
>44.6	475(16.6%)	358(17.9%)	117(13.7%)	
**HGB (g/L)**				0.220
≤127	1375(48.1%)	948(47.4%)	427(49.9%)	
>127	1482(51.9%)	1053(52.6%)	429(50.1%)	
**TRF (g/L)**				**0.024**
≤2.25	684(23.9%)	455(22.7%)	229(26.8%)	
>2.25	2173(76.1%)	1546(77.3%)	627(73.2%)	
**TP (g/L)**				0.094
≤70.2	1406(49.2%)	964(48.2%)	442(51.6%)	
>70.2	1451(50.8%)	1037(51.8%)	414(48.4%)	
**AGR(g/L)**				0.091
≤1.66	2480(86.8%)	1751(87.5%)	729(85.2%)	
>1.66	377(13.2%)	250(12.5%)	127(14.8%)	
**PA (mg/L)**				0.067
≤216	924(32.3%)	626(31.3%)	298(34.8%)	
>216	1933(67.7%)	1375(68.7%)	558(65.2%)	
**SF (μg/L)**				1.000
≤240	2374(83.1%)	1674(83.7%)	700(81.8%)	
>240	483(16.9%)	372(16.3%)	156(18.2%)	
**LDH (U/L)**				0.741
≤212	2387(83.5%)	1675(83.7%)	712(83.2%)	
>212	470(16.5%)	326(16.3%)	144(16.8%)	

Bold values indicate P-values < 0.05.

### 3.2 Factors correlated with plasma levels of LYM, PLT, AGR, PA and HGB in BC patients with their interrelationship

**Figure 2:**
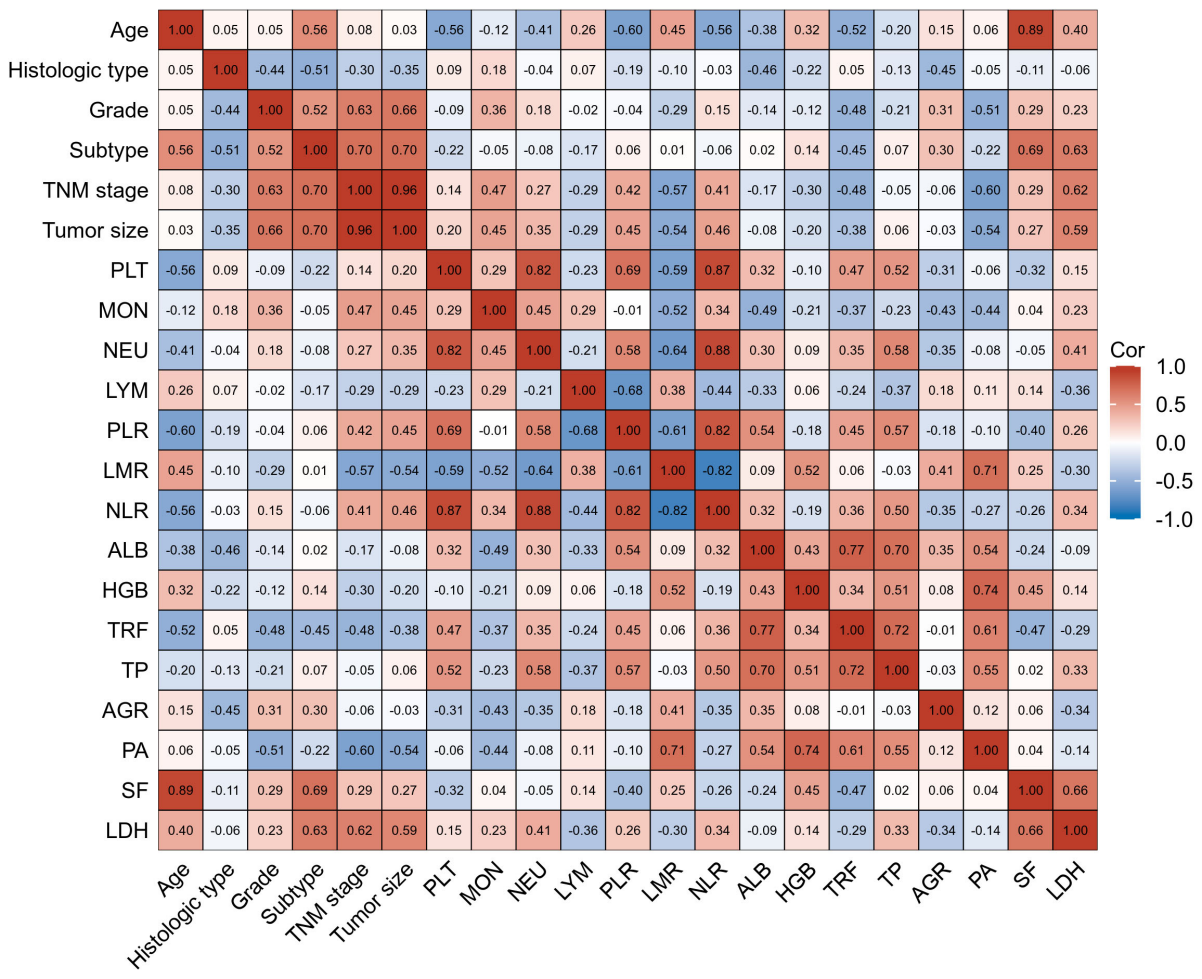
Factors correlated with plasma levels of LYM, PLT, AGR, PA, and HGB in training cohort BC patients with their interrelationship. The heat map showing a red background indicates a positive correlation, and the blue background indicates a negative correlation.

The interrelationships between plasma levels of LYM, PLT, AGR, PA, HGB and clinical factors in BC patients are presented in **Figure 2**. AGR levels showed a correlation with age and subtype, indicating that AGR levels were influenced by these factors. LYM levels, on the other hand, only showed a correlation with age. Interestingly, HGB and PA levels did not show significant correlations with age or subtype. Furthermore, PLT levels were found to be negatively correlated with age, suggesting that PLT levels decrease as age increases. PA levels, on the other hand, were negatively correlated with TNM stage, and tumor size, indicating that higher PA levels were associated with less advanced disease.

In terms of interrelationships among the biomarkers, PLT levels were not correlated with HGB levels. However, they were negatively correlated with LYM, PA, and AGR levels, indicating that higher PLT levels were associated with lower LYM, PA, and AGR levels. Additionally, AGR, PA, and HGB levels showed correlations with LMR, suggesting a potential relationship between these biomarkers and LMR.

These findings provide insights into the interrelationships between plasma biomarker levels and various clinical factors in BC patients, highlighting their potential as prognostic indicators and contributing to our understanding of the disease.

### 3.3 Univariate analyses and multivariate analyses

The univariate analysis of potential factors associated with BC revealed significant associations with the following factors: age (*p*=0.005), subtype (*p <*0.001), TNM stage (*p*<0.001), tumor size (*p*<0.001), PLT (*p*<0.001), MON (*p*<0.001), NEU (*p*<0.001), LYM (*p*=0.048), LMR (*p*=0.001), NLR (*p*=0.002), HGB (*p*=0.007), TRF (*p*=0.001), AGR (*p*=0.002), PA (*p*<0.001), SF (*p*<0.001), and LDH (*p*<0.001). Based on these significant factors, a multivariate analysis was conducted. The results showed that the following factors were significantly associated with BC: subtype (HR=6.461; 95% CI=2.860~14.595; *p*<0.001), TNM stage (HR=9.603; 95% CI=4.080~22.602; *p*<0.001), PLT (HR=1.374; 95% CI=1.016~1.860; *p* =0.039), LYM (HR=1.748; 95% CI=1.043~2.930; *p* =0.034), HGB (HR= 0.690; 95% CI=0.510~0.935 *p* =0.017), AGR (HR=1.951; 95% CI=1.389~2.740; *p <*0.001), and PA (HR= 0.694;95% CI=0.507~0.949; *p* =0.022). The detailed univariate and multivariate analysis results are presented in **Table 2**.

**Table 2:** Univariate and multivariate analysis of OS in the training cohort.

Characteristic	Univariate analysis	*p*	Multivariate analysis	*p*
	HR (95% CI)		HR (95% CI)	
Age (years)				
≤52 vs >52	1.513(1.135~2.018)	0.005	1.349(0.967~1.882)	0.078
**Histologic type**		0.789		
IDC vs ILC	1.295(0.573~2.927)	0.535		
IDC vs Others	0.955(0.660~1.380)	0.805		
**Grade**		0.150		
I vs II	1.100(0.693~1.744)	0.687		
I vs III	1.412(0.903~2.208)	0.130		
**Subtype**		<0.001		**<0.001**
Luminal A vs Luminal B	3.663(1.707~7.862)	0.001	3.291(1.527~7.091)	0.002
Luminal A vs Her-2	5.901(2.640~13.193)	<0.001	3.671(1.626~8.290)	0.002
Luminal A vs TNBC	7.544(3.357~16.953)	<0.001	6.461(2.860~14.595)	<0.001
**TNM stage**		<0.001		**<0.001**
I vs II	1.850(0.992~3.452)	0.053	1.013(0.441~2.327)	0.975
I vs III	7.555(4.110~13.889)	<0.001	3.501(1.540~7.959)	0.003
I vs IV	24.867(13.236~46.717)	<0.001	9.603(4.080~22.602)	<0.001
Tumor size (cm)				
≤2 vs >2	3.427(2.235~5.255)	<0.001	1.654(0.909~3.010)	0.099
PLT (10^9^/L)				
≤288.56 vs >288.56	1.739(1.311~2.307)	<0.001	1.374(1.016~1.860)	**0.039**
MON (10^9^/L)				
≤0.39 vs >0.39	1.687(1.272~2.236)	<0.001	1.292(0.912~1.831)	0.149
NEU (10^9^/L)				
≤4.84 vs >4.84	1.976(1.466~2.662)	<0.001	1.385(0.966~1.984)	0.076
LYM (10^9^/L)				
≤1.37 vs >1.37	1.631(1.004~2.650)	0.048	1.748(1.043~2.930)	**0.034**
PLR (10^9^/L)				
≤196.4 vs >196.4	1.340(0.960~1.871)	0.085		
LMR (10^9^/L)				
≤4.92 vs >4.92	0.611(0.460~0.811)	0.001	0.976(0.676~1.411)	0.899
NLR (10^9^/L)				
≤1.69 vs >1.69	1.680(1.212~2.328)	0.002	1.166(0.795~1.710)	0.433
ALB (g/L)				
≤44.6 vs >44.6	1.230(0.884~1.712)	0.219		
HGB (g/L)				
≤127 vs >127	0.676(0.509~0.898)	0.007	0.690(0.510~0.935)	**0.017**
TRF (g/L)				
≤2.25 vs >2.25	0.611(0.453~0.826)	0.001	0.765(0.551~1.062)	0.109
TP(g/L)				
≤70.2 vs >70.2	1.197(0.898~1.594)	0.220		
A/G(g/L)				
≤1.66 vs >1.66	1.693(1.210~2.367)	0.002	1.951(1.389~2.740)	**<0.001**
PA (mg/L)				
≤216 vs >216	0.592(0.445~0.786)	<0.001	0.694(0.507~0.949)	**0.022**
SF (μg/L)				
≤240 vs >240	2.004(1.461~2.748)	<0.001	1.254(0.872~1.803)	0.221
LDH(U/L)				
≤212 vs >212	2.620(1.937~3.543)	<0.001	1.292(0.907~1.840)	0.155

### 3.4 Construction and validation of the nomogram

**Figure 3:**
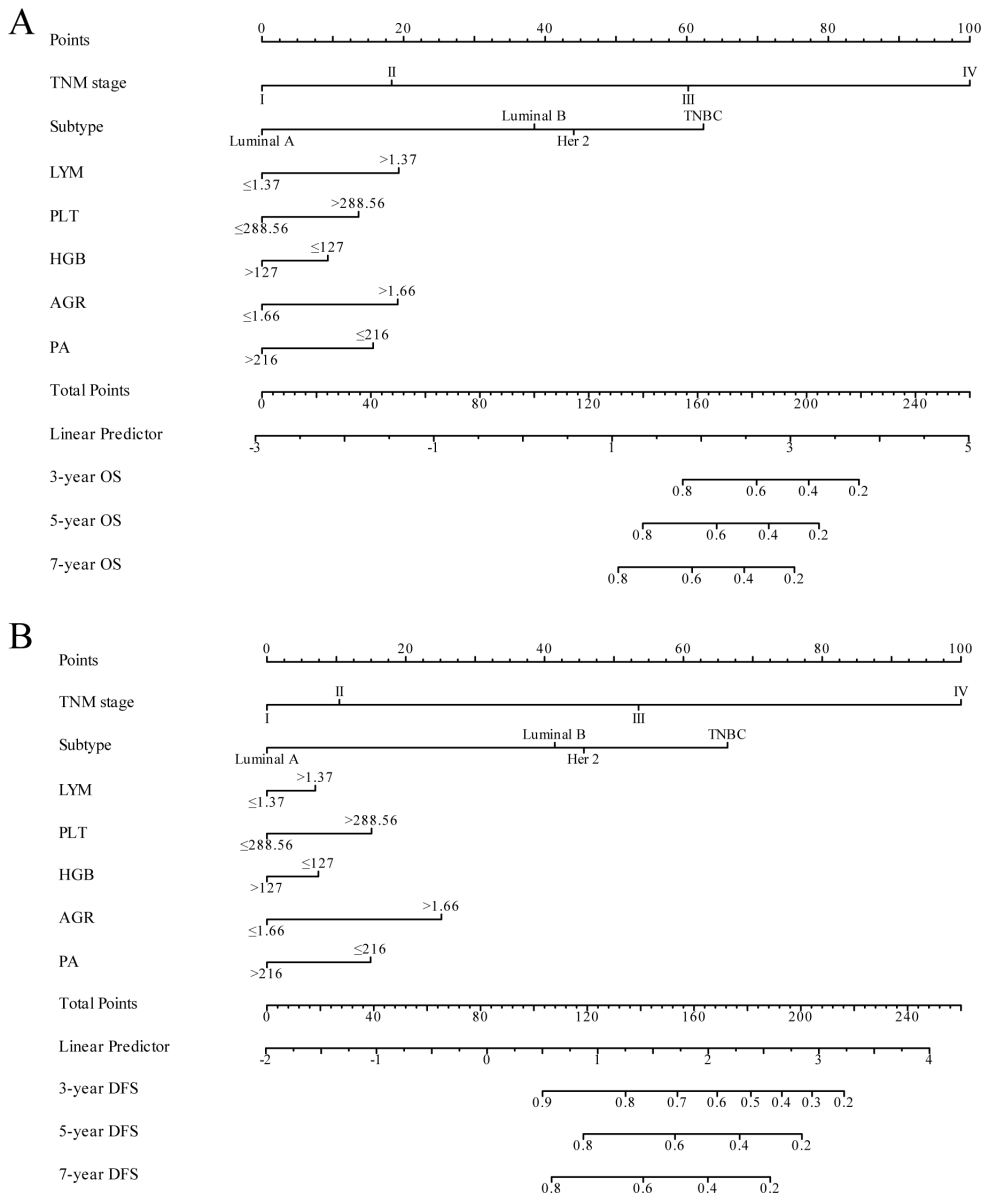
Nomogram model for predicting 3-year, 5-year and 7-year OS **(A)** and DFS **(B)** in BC patients within the training cohort. The nomogram was used to sum the points identified on the points scale for each variable. The total points projected on the bottom scales indicate the probability of 3-, 5- and 7-year survival.

**Figure 4:**
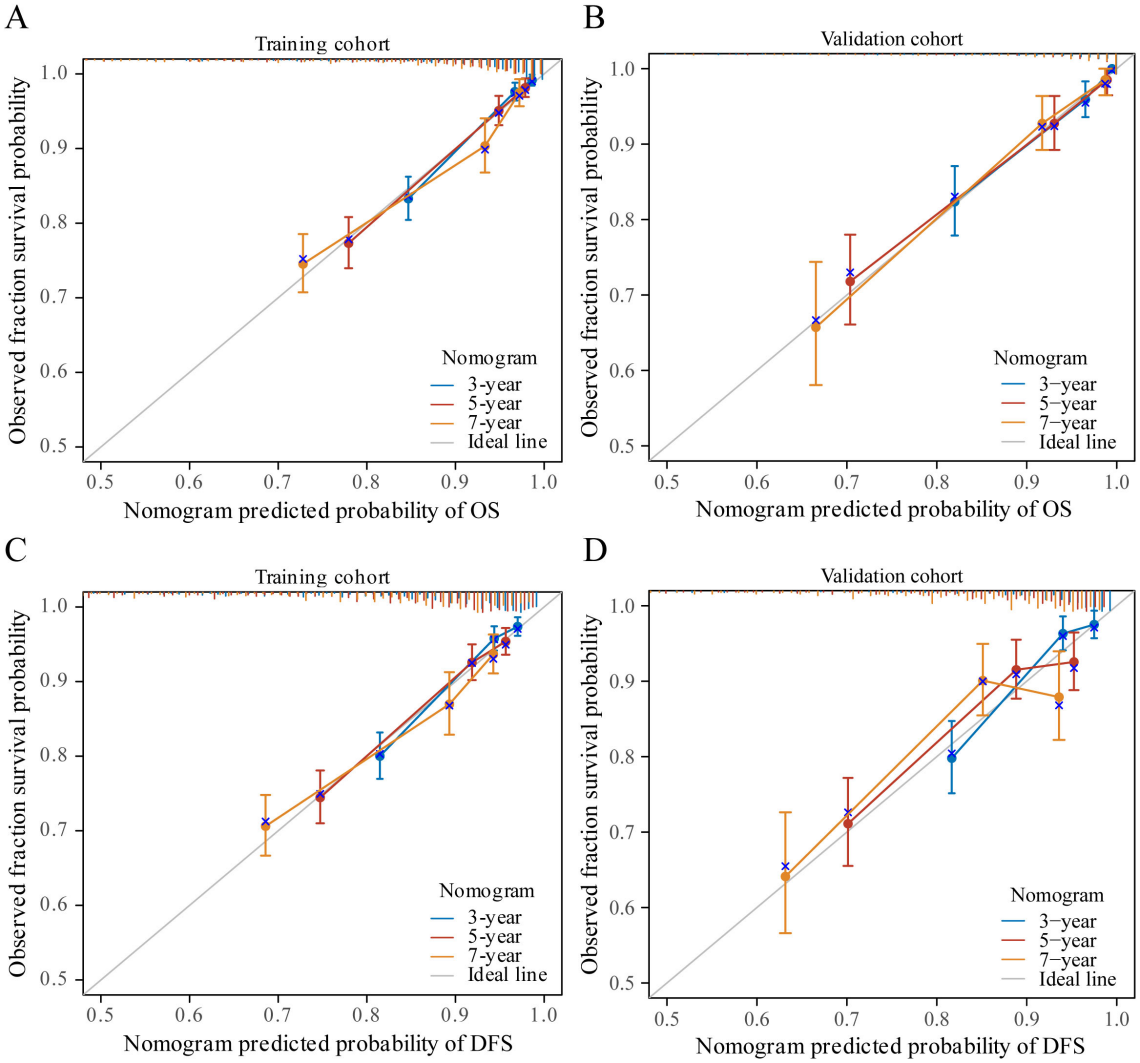
The calibration curves for predicting patient OS and DFS at three years, five years and seven years in the training cohort **(A, C)** and at three years, five years and seven years in the validation cohort **(B, D)**.

In our study, TNM stage, subtype, LYM, PLT, HGB, AGR, and PA indicators, along with their respective cutoff values, performed well in the OS model, we have chosen to apply these indicators and cutoff values to the DFS model. The purpose of this is to ensure that our DFS model maintains reliable predictive performance and consistency with the OS model. The nomogram, based on the multivariate analysis results from the training cohort, was constructed to predict OS as well as DFS in BC patients. The nomogram incorporated all the independent prognostic factors identified in the multivariate analysis, including TNM stage, subtype, PLT, LYM, HGB, AGR, and PA. **Figure 3** represents the nomogram for the training cohort. It is straightforward to estimate the 3-year, 5-year and 7-year OS and DFS probabilities by summing the scores associated with each variable and projecting the sums to the bottom scales. The model predicted the OS and DFS rates of BC with high accuracy in the training cohort, as indicated by a C-index of 0.820 (95% CI, 0.805–0.835) for OS and 0.760 (95% CI, 0.744–0.776) for DFS. In the training cohort, the calibration plot in **Figures 4A, C** shows a strong correlation between the predicted probabilities of 3-year, 5-year, and 7-year OS and DFS from the nomogram and the actual observed survival rates after surgery, indicating a high degree of concordance. Similarly, consistent results were observed in the validation cohort. The C-index of the nomogram for predicting OS and DFS in the validation cohort was 0.838 (95% CI, 0.818–0.858) and 0.755 (95% CI, 0.730–0.780), respectively. In the validation cohort, the calibration plot in **Figures 4B, D** also demonstrates good consistency between the predicted and actual OS and DFS. These findings imply that a nomogram is an accurate method for estimating survival in BC patients and offers useful data for clinical decision-making and patient counseling.

### 3.5 Risk stratification of OS and DFS

**Figure 5:**
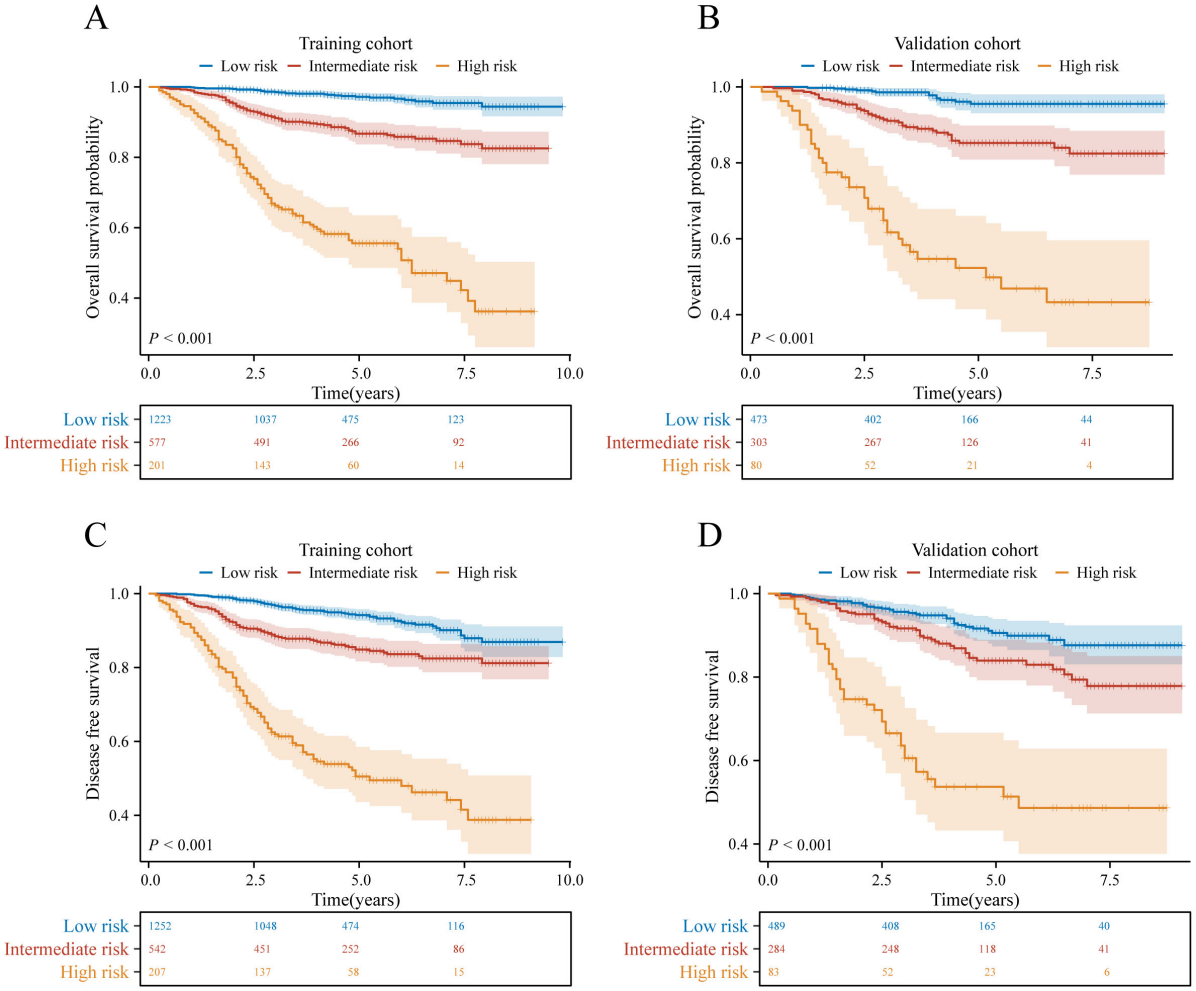
Graphs showing the results of Kaplan–Meier curves for all three groups based on the predictor from the nomogram model in the training cohort **(A, C)** and those in the validation cohort **(B, D)**.

The X-tile program was used to determine total point thresholds, based on which patients in both the training and validation cohorts were divided into low-, intermediate-, and high-risk groups for both OS and DFS. In the training cohort, the OS rates for the low-risk, intermediate-risk, and high-risk groups were 97.5%, 87.5%, and 54.7%, respectively (*p*<0.001, **Figure 5A**), while the DFS rates for the training cohort were 94.3%, 85.2%, and 51.6%, respectively (*p*<0.001, **Figure 5C**). Similarly, in the validation cohort, the OS rates for these risk categories were 97.3%, 86.8%, and 53.8%, respectively (*p*<0.001, **Figure 5B**), and the DFS rates were 92.6%, 84.8%, and 55.4%, respectively (*p*<0.001, **Figure 5D**). This risk stratification accurately determined survival outcomes for the three different categories within the training and validation cohorts.

### 3.6 Comparison of predictive accuracy and clinical usability between nomogram and TMN staging systems

**Figure 6:**
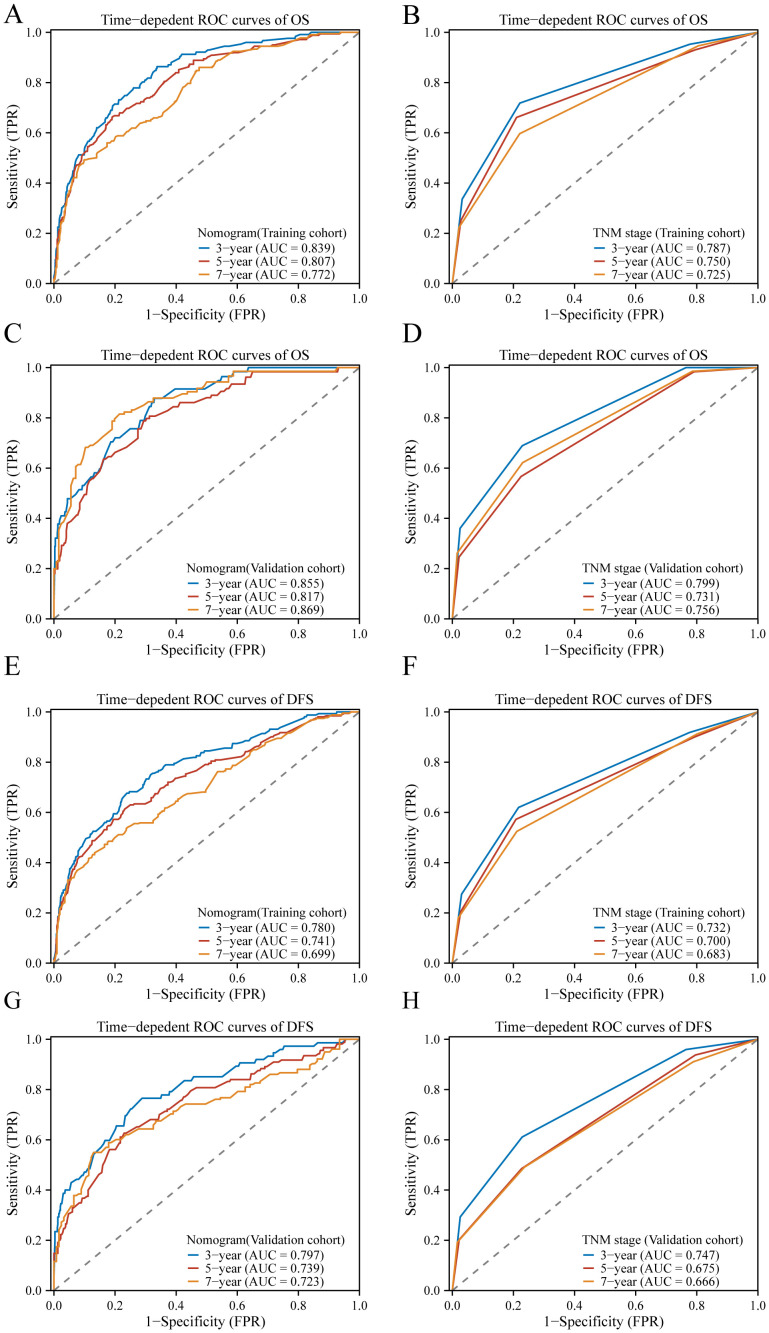
The ROC curves of the model to predict BC OS and DFS at 3, 5, and 7 years; In the training cohort, ROC curves comparing the nomogram **(A)** and TNM stage **(B)** for predicting OS, and in the validation cohort **(C, D)**, respectively. Similarly, in the training cohort, ROC curves compare the nomogram **(E)** and TNM stage **(F)** for predicting DFS, and in the validation cohort **(G, H)**, respectively.

ROC analysis further confirmed the superiority of the nomogram over the TNM stage model, exhibiting higher AUC values across various time points for both OS and DFS. Specifically, in the training cohort, the nomogram achieved AUC values of 0.839, 0.807, and 0.772 for 3-year, 5-year, and 7-year OS, respectively (**Figure 6A**), compared to AUC values of 0.787, 0.750, and 0.725 for the TNM stage model (**Figure 6B**). Similarly, for DFS, the nomogram achieved AUC values of 0.780, 0.741, and 0.699 for 3-year, 5-year, and 7-year DFS, respectively (**Figure 6E**), compared to AUC values of 0.732, 0.700, and 0.683 for the TNM stage model in the training cohort (**Figure 6F**). These trends were consistent in the validation cohort, where the nomogram demonstrated higher AUC values for both OS and DFS at each time point compared to the TNM stage model (**Figures 6C, D, G, H**). The ROC curves depicted in **Figure 6** further illustrate the enhanced predictive performance of the nomogram over the TNM stage model. In summary, these findings indicate that the nomogram provides superior predictive accuracy and clinical usability in forecasting survival outcomes across different time intervals.

In the training cohort, the C-index of the nomogram was higher than the C-index of the TMN stage, Subtype, and TMN stage + Subtype, respectively. Similarly, consistent results were observed in the validation cohort. The results are shown in **Table 3**. These findings provide insights into the interrelationships between plasma biomarker levels and various clinical factors in BC patients, highlighting inflammation and nutritional biomarker’s potential as prognostic indicators and contributing to predicting the survival of BC patients.

**Table 3:** The C-indexes of nomograms, TNM stage, Subtype, and TNM stage+ Subtype for prediction of OS and DFS in the training cohort and validation cohort.

	Training cohort		Validation cohort	
For OS	C-index	HR (95% CI)	C-index	HR (95% CI)
**Nomograms**	0.82	(0.805–0.350)	0.838	(0.818–0.858)
**TNM stage**	0.759	(0.741–0.778)	0.772	(0.749–0.794)
**Subtype**	0.628	(0.610–0.647)	0.611	(0.585–0.637)
**TMN stage + Subtype**	0.801	(0.785–0.817)	0.802	(0.779–0.825)
For DFS				
**Nomograms**	0.76	(0.744–0.776)	0.755	(0.730–0.780)
**TNM stage**	0.707	(0.689–0.725)	0.708	(0.684–0.732)
**Subtype**	0.611	(0.595–0.627)	0.583	(0.560–0.607)
**TMN stage + Subtype**	0.744	(0.728–0.761)	0.728	(70.703–0.754)

**Figure 7:**
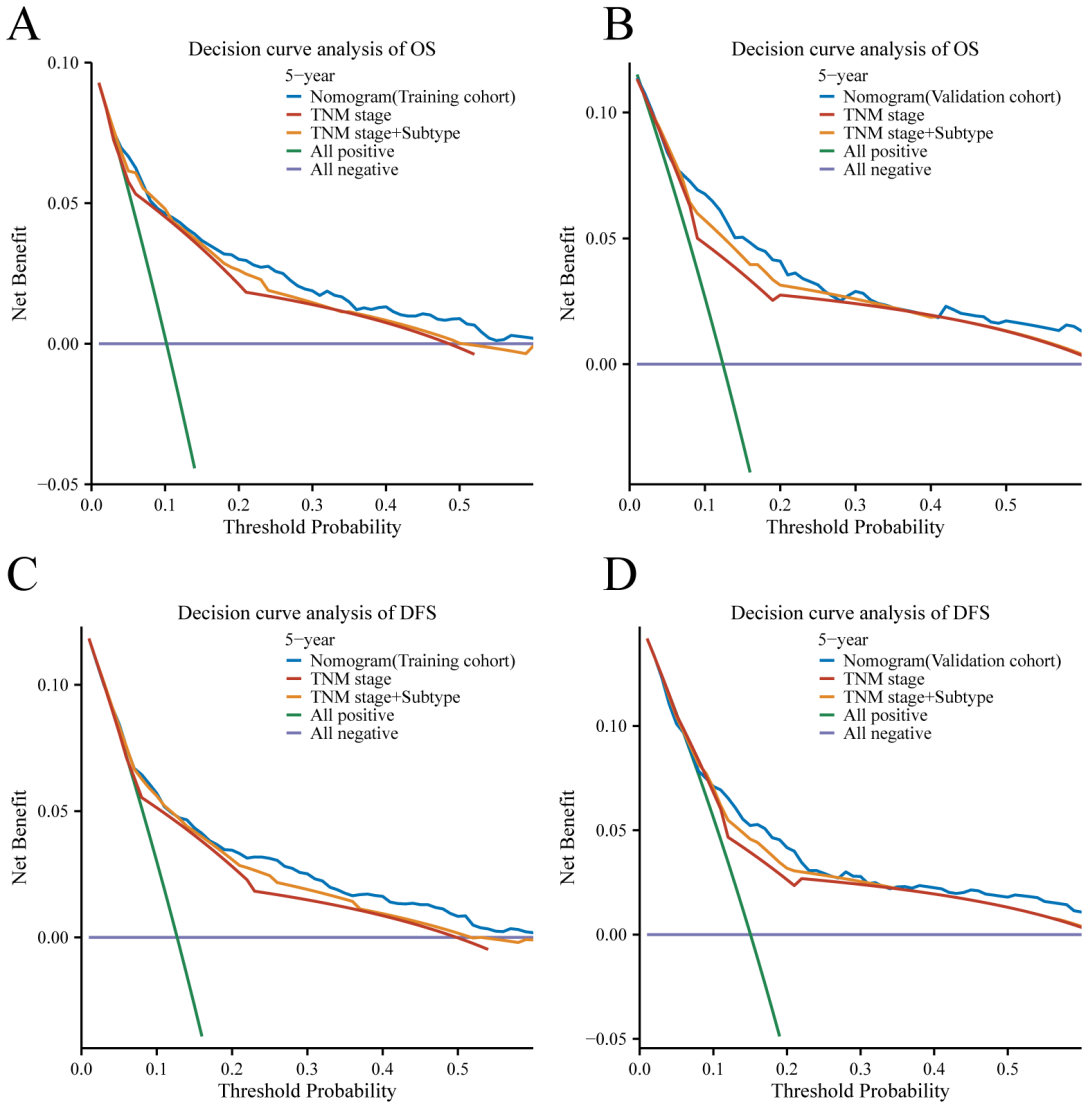
Decision curve analysis for 5-year survival predictions. **(A, C)** The decision curve of the training cohort; **(B, D)** The decision curve of the validation cohort.

To compare the clinical utility of this approach with traditional TNM staging, a decision curve analysis (DCA) was carried out. The DCA displayed graphically the net benefit of using the nomogram and TNM stage model to predict 5-year OS and DFS in the training and validation cohorts, taking into account a range of various recurrence threshold probabilities on the x-axis. The DCA plots (**Figure 7**) demonstrated that the nomogram provided a greater net benefit than other prognostic factors including the TNM stage nomogram model across the range of threshold probabilities evaluated. The nomogram showed higher net benefit curves, indicating that using the nomogram for risk stratification resulted in a higher overall net benefit in predicting 5-year OS and DFS. This suggests that the nomogram has a greater clinical utility than the conventional TNM stage model, as it provides superior risk stratification and enhances clinical decision-making. In summary, the DCA results further support the superiority of the nomogram over the TNM stage model in terms of clinical usefulness, as it offers greater net benefit in predicting 5-year OS and DFS across a range of threshold probabilities for recurrence.

### 3.7 External validation of the nomogram

**Figure 8:**
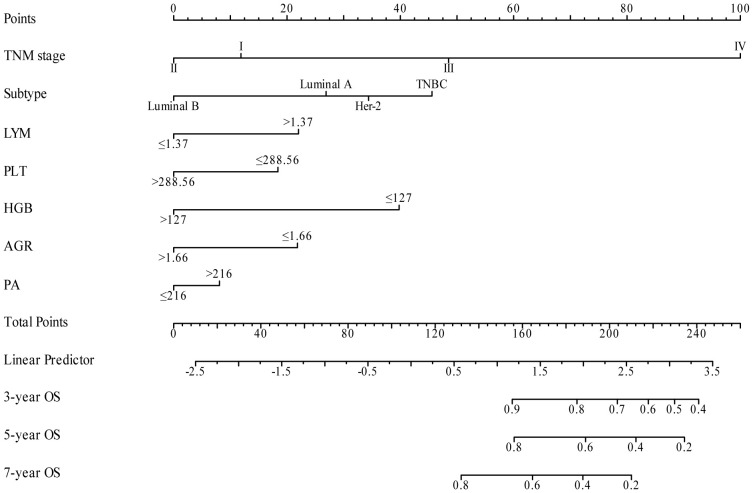
Nomogram model for predicting 3-year, 5-year, and 7-year OS in BC patients within the external validation cohort.

The external validation cohort, consisting of 420 cases, was utilized as an independent validation set to assess the performance of our nomogram model. The C-index of the nomogram in this validation cohort was found to be 0.772 (95% CI: 0.726–0.817) (**Figure 8**). The validation of a model with a C-index exceeding 0.7 suggests that the model constructed in this study performs well in terms of robustness and reliability, thereby enhancing the credibility and persuasiveness of our research findings.

## 4 Discussion

In the present study, we constructed and confirmed a nomogram model that combines easily accessible inflammation and nutritional factors, and clinicopathological variables to predict OS and DFS in BC patients. The nomogram presented superior predictive accuracy, discriminative ability, and clinical usefulness compared to the traditional TNM stage system. Clinicians can utilize this nomogram to guide treatment decisions, monitor disease progression, and provide personalized patient care.

A growing body of research has demonstrated that inflammation and nutritional status play important roles in both tumor development and patient prognosis (39, 40). Inflammatory responses play a key role in the tumor microenvironment in regulating tumor growth, metastasis and treatment resistance (12). Nutritional status directly affects the immune function, metabolic status and physiological regulation of the patient, resulting in their resistance to tumor and therapeutic response (41). This study found that inflammatory (LYM, PLT) and nutritional (HGB, AGR, PA) indicators were strongly associated with BC prognosis. Watanabe J et al. and Kazuhiro Araki et al. discovered that elevated LYM levels are associated with an improved response to chemotherapies in metastatic BC patients (42, 43). Sung Min Ko et al. reported that LYM was a strong predictor of DFS in BC patients (44), which is consistent with our results. LYM plays a crucial role in anti-tumor immunity by inducing tumor cell apoptosis and is one of the key factors in immune surveillance and immune editing (45, 46).They combat tumors through multiple mechanisms, including the activation of cytotoxic T cells and natural killer (NK) cells, which directly target and eliminate tumor cells. Additionally, lymphocytes secrete cytokines such as interferon-gamma (IFN-γ and tumor necrosis factor-alpha (TNF-α which enhance the anti-tumor activity of other immune cells. These cytokines modulate the tumor microenvironment by promoting an immune-activating milieu and inhibiting tumor growth and metastasis (47, 48). Some studies have also shown that a low LYM may be the cause of inadequate immune response and the result of low survival rates in many types of cancer. This immune deficiency may lead to increased tumor proliferation and metastasis and reduced response to therapeutic interventions (49). Therefore, the immune response to breast tumors could vary depending on the composition of lymphocytes, which ultimately affects prognosis. Further research is needed on the mechanism of peripheral lymphocytes affecting BC. Discoveries have uncovered that PLTs function in inflammatory diseases and malignant tumors (50, 51). PLTs are closely associated with tumor cells and play a crucial role in the key stage of cancer metastasis. PLT regulates immune responses in the tumor microenvironment by secreting a variety of cytokines and growth factors (e.g., transforming growth factor-β, TGF-β). TGF-β not only inhibits the proliferation and function of lymphocytes, weakening the body’s immune surveillance and immune editing ability but also promotes the growth and invasion of tumor cells, thus inhibiting the anti-tumor activity of immune cells (52). Through the release of cytokines, tumor cells activate platelets, promoting the extravasation and spread of cancer cells and negatively correlates with survival prognosis (53, 54). Notably, in our study, platelets were an independent prognostic factor of BC, which was also confirmed by the study of Liefaard, M. C. et al. and Graziano, V. et al (55, 56). Changes in LYM and platelets PLT, as indicators of inflammation, not only directly affect the immune response, but also indirectly influence tumor progression by affecting the nutritional status. Our research indicates that HGB is a potential prognostic indicator for BC. A study by Michael Henke et al. showed that HGB concentration affects the prognosis of patients with early BC, which corresponds to our results (57). Several investigations indicated anemia and HGB play a pivotal role in malignant progression (58, 59). An important factor contributing to tumor hypoxia is the reduced oxygen transport capacity in the blood resulting from tumor-related and/or treatment-related anemia, which is a frequent complication seen in cancer patients (59, 60). HGB is an important prognostic indicator of nutritional status and hypoxia in cancer patients. Hence, it is essential to elucidate the underlying mechanisms of HGB that affect BC. In cancer patients, changes in the AGR are closely related to prognosis (61). Cancer-related chronic inflammation and malnutrition typically lead to a decrease in AGR, primarily by reducing albumin levels and increasing globulin levels. A low AGR usually indicates poor prognosis, higher recurrence rates, and shorter survival times (62). In the present study, few studies have systematically examined the connection between BC and AGR (63, 64). Basem N. Azab has shown that pretreatment AGR is an independent, significant predictor of long-term mortality in BC patients (65). Additionally, this research demonstrates that AGR is an independent predictive factor for BC. PA, also known as transthyretin, is a thyroid hormone transport protein synthesized by the liver and partially degraded by the kidneys; its primary function is thyroxine transport. Serum PA concentrations less than 10 mg/dL are associated with malnutrition (66). PA is more sensitive to acute changes in protein balance and reacts to nutrition (67, 68). Numerous studies indicate that PA is a helpful single parameter for assessing protein-energy malnutrition, including postoperative outcomes and recurrence of non-small cell lung cancer (69, 70). Our research also revealed the prognostic significance of PA in BC. We have determined a comprehensive and systematic assessment, combining inflammation and nutritional blood markers, to evaluate their impact on the prognosis of BC.

Our nomogram demonstrated a significant improvement in predicting OS and DFS of BC patients compared to the TNM stage system. The model was further validated in an independent external cohort, confirming its reliability and reproducibility. Currently, several prognostic models are accessible for the clinical assessment of BC patients (71). Jeongmin Lee et al. and Xuanyi Wang et al. both developed a prognostic model based on radiomics to predict the DFS in BC patients, achieving a C-index of 0.63 and 0.82, respectively (72, 73). There are also nomograms based on molecular testing, gene expression profiling, and RNA sequencing data that can provide accurate predictions for BC patients. For instance, Jie Sun et al. created a model to predict BC risk in BRCA gene carriers, but in an empirical investigation, their C index was only 0.711 (74). MammaPrint test on 70 genes proved useful for early-stage BC treatment decisions (75), the C-index of the model for predicting OS was 0.614 (76). Liu Z. et al. developed a nomogram composed of 7-lncRNA signatures associated with immune invasion and tumor mutation burden in BC (77). However, molecular testing, gene expression profiling, and RNA sequencing data were not included in our model due to their requirement for highly specialized testing facilities, which entail high costs and necessitate skilled personnel for operation, thereby limiting their applicability. Our nomogram achieved a C-index of 0.820 for OS and 0.76 for DFS in the training cohort, which is relatively high compared to the analyses described above. Notably, in our study, the acquisition of hematological indicators is generally non-invasive, simpler, and more cost-effective, making them suitable for dynamic monitoring. These indicators can reflect changes in the patient’s condition and provide comprehensive information on systemic status, including inflammatory responses, immune function, and nutritional status. In contrast, radiomics may involve the use of radiation or contrast agents, posing certain risks and discomfort. Moreover, radiomics can only provide structural information for specific sites and typically requires longer intervals between repeated assessments. Our prediction models can be cheaper, more accurate, and simpler to use in primary hospitals compared to these models. We developed a nomogram to predict 3-year, 5-year, and 7-year OS and DFS for BC patients in both training and validation cohorts. This tool aids clinicians in estimating individual survival probabilities with greater precision. Our nomogram demonstrates better prognostic accuracy and clinical utility. This prognostic model can be of great clinical value for patient management, risk stratification, therapeutic options, and postoperative monitoring strategies.

Despite the excellent discrimination ability of our nomogram, our research has its limitations. First, as with any retrospective study analysis, there is a potential risk of selection bias. Second, despite the use of an independent external validation cohort in this study, further research involving a multi-center prospective study with a larger dataset is warranted. Third, due to the limitations of our database, we are unable to incorporate genetic variables such as BRCA1/2 and P53 into our current model. We plan to explore and collect additional genetic and molecular marker data in future research to improve predictive accuracy and provide deeper insights into the biological complexity of BC.

## 5 Conclusion

Our study successfully developed a predictive nomogram for OS and DFS in BC patients by incorporating inflammation, nutritional factors, and pathologic, which showed greater precision than the conventional TNM staging system. The nomogram in this study was validated using independent cohorts from different institutions. Independent validation of the model with a C-index greater than 0.7 indicates that our model exhibits good performance in terms of robustness and reliability. The nomogram is a straightforward, low-cost, and useful tool that can assist clinicians with choosing therapies and patient counseling. Further analysis and validation studies are warranted to refine and improve the nomogram, taking into account the limitations mentioned above, and to establish its usefulness in clinical practice.

## Data availability statement

The original contributions presented in the study are included in the article/supplementary material. Further inquiries can be directed to the corresponding authors.

## Ethics statement

The studies involving humans were approved by Guangxi Medical University Cancer Hospital Ethical Review Committee; Guangxi Zhuang Autonomous Region Maternal and Child Health Care Hospital Ethical Review Committee. The studies were conducted in accordance with the local legislation and institutional requirements. The participants provided their written informed consent to participate in this study.

## Author contributions

CW: Writing – original draft, Investigation, Methodology, Software. HA: Software, Writing – original draft, Data curation, Formal analysis, Validation. DM: Methodology, Supervision, Visualization, Writing – original draft. PW: Formal analysis, Resources, Writing – original draft, Data curation. LW: Formal analysis, Resources, Writing – original draft. ZL: Writing – original draft, Validation. PL: Validation, Writing – original draft. TH: Writing – original draft, Visualization, Writing – review & editing. ML: Writing – original draft, Writing – review & editing, Conceptualization.
